# Health care seeking behaviours in pregnancy in rural Sindh, Pakistan: a qualitative study

**DOI:** 10.1186/s12978-016-0140-1

**Published:** 2016-06-08

**Authors:** Rahat Najam Qureshi, Sana Sheikh, Asif Raza Khowaja, Zahra Hoodbhoy, Shujaat Zaidi, Diane Sawchuck, Marianne Vidler, Zulfiqar A. Bhutta, Peter von Dadeslzen, CLIP Working Group

**Affiliations:** Division of Women & Child Health, Aga Khan University, Karachi, Pakistan; Department of Obstetrics and Gynaecology, and the Child and Family Research Institute, University of British Columbia, Vancouver, Canada; Program for Global Pediatric Research, Hospital for Sick Children, Toronto, Canada

**Keywords:** Health seeking behaviour, Pregnancy, Antenatal care, Associated factors, Pakistan, Health facilities, Patient acceptance of health care, Pregnancy complications

## Abstract

**Background:**

Pakistan has alarmingly high numbers of maternal mortality along with suboptimal care-seeking behaviour. It is essential to identify the barriers and facilitators that women and families encounter, when deciding to seek maternal care services. This study aimed to understand health-seeking patterns of pregnant women in rural Sindh, Pakistan.

**Methods:**

A qualitative study was undertaken in rural Sindh, Pakistan as part of a large multi-country study in 2012. Thirty three focus group discussions and 26 in-depth interviews were conducted with mothers [*n* = 173], male decision-makers [*n* = 64], Lady Health Workers [*n* = 64], Lady Health Supervisors [*n* = 10], Women Medical Officers [*n* = 9] and Traditional Birth Attendants [*n* = 7] in the study communities. A set of a priori themes regarding care-seeking during pregnancy and its complications as well as additional themes as they emerged from the data were used for analysis. Qualitative analysis was done using NVivo version 10.

**Results:**

Women stated they usually visited health facilities if they experienced pregnancy complications or danger signs, such as heavy bleeding or headache. Findings revealed the importance of husbands and mothers-in-law as decision makers regarding health care utilization. Participants expressed that poor availability of transport, financial constraints and the unavailability of chaperones were important barriers to seeking care. In addition, private facilities were often preferred due to the perceived superior quality of services.

**Conclusion:**

Maternal care utilization was influenced by social, economic and cultural factors in rural Pakistani communities. The perceived poor quality care at public hospitals was a significant barrier for many women in accessing health services. If maternal lives are to be saved, policy makers need to develop processes to overcome these barriers and ensure easily accessible high-quality care for women in rural communities.

**Trial Registration:**

NCT01911494

**Electronic supplementary material:**

The online version of this article (doi:10.1186/s12978-016-0140-1) contains supplementary material, which is available to authorized users.

## Background

Appropriate and timely care seeking is essential for healthy outcomes for individuals and communities [1]. Understanding health seeking behaviour in a community is necessary for the development of appropriate health policies, health systems and educational strategies to facilitate access. The decision to seek care is complex and driven by many determinants [1]. The behaviour may differ depending on the nature of the complaint as well as the social circumstances of the patient [2].

Behavioural models can be used to illustrate the dynamics of the individual, community and care providers in such decisions [1]. Andersen’s behavioural model describes three categories of determinants at play in health care decision making: predisposing characteristics, enabling characteristics, and need [1]. Predisposing characteristics include the individual’s demographics, their social structure as well as their beliefs regarding the benefits of health services. Enabling characteristics encompass personal and community resources that encourage usage of health services. Finally, the third category relates to the perceived and actual need for services.

Theories used to explain health care utilization assume that the recognition of symptoms suggests illness; however, in regions with poor living conditions such as temperature extremes, lack of potable water and fuel, women’s perception of feeling unwell may be unique [3], particularly when they have low social status and their sense of ‘feeling unwell’ may not be validated by family members [4].

During pregnancy, the choice of provider and facility is often dependent on women’s social and physical environment. Women with greater autonomy, which is measured by control over finances, decision making power and freedom of movement, have a greater preponderance for accessing care during pregnancy [5]. Health services may be accessed for routine antenatal care, for delivery, for treatment, or emergencies.

The Pakistan Demographic and Health Survey (PDHS) 2006–07 revealed that only 65 % of women seek routine care during pregnancy, and amongst these, 4 % seek care outside the formal health system. Sixty five percent of women delivered at home, and only 39 % of deliveries took place in presence of a skilled birth attendant (doctor, nurse, midwife or lady health visitor) [6]. Postpartum care was less common than antenatal care (43 vs. 61 % respectively) [6].

Pakistan is the 6th most populous country and given the current fertility rate it is likely to maintain this position [7]. Overall 65 % of the population resides in rural areas. Many have limited education and only 24 % of women and 65 % of men have formal education. In addition, household conditions are often substandard, where only 22 % of households have piped water, and 88 % of households have no latrine connected to a piped sewerage system. Living conditions are particularly challenging in Sindh province where average temperatures are 30–40 ^°^C [8].

Most rural homes are at least 10 km from the district hospital or maternal and child health centre. The most readily available health care providers in these communities are traditional birth attendants (TBA), alternative medicine practitioners or pharmacists [6]. The health care system of Pakistan comprises of public and private sectors. The public sector delivers services at the primary, secondary and tertiary level. The primary level includes rural health centres, basic health units, primary health care centres, dispensaries, first aid posts, mother and child health centres, and community health workers known as Lady Health Workers (LHW). The secondary level includes the district and Tehsil Headquarter hospitals whereas the tertiary level care is delivered through the teaching hospitals. The private sector consists of allopathic as well spiritual/traditional healers [9].

Pakistan has been unable to achieve a substantial reduction in the maternal mortality ratio (MMR), the World Health Organization (WHO) estimated the MMR in Pakistan at 260 deaths per 100,000 live births [10]. Although there has been progress in the reduction of maternal mortality, trends indicate that Pakistan lags behind many South Asian countries. Haemorrhage, pre-eclampsia and puerperal sepsis account for 57 % of maternal deaths in Pakistan [6, 7]. The vast majority of these deaths could be prevented through access to basic antenatal care services [10]. Inadequate access and under-utilization of the health care system are major factors for poor health indicators of developing countries [10]. These numbers reflect the suboptimal care seeking behaviour during pregnancy in Pakistani women.

The aim of this study was to assess the perception of pregnant women and their families regarding health seeking behavior during pregnancy and its complications, and to evaluate the barriers and facilitators during this process.

## Methods

A qualitative study was undertaken between February-July 2012 in Pakistan, as part of a large multi-country study, a detailed description of the methods has been published. This study served as the formative research for an upcoming international trial, Community Level Interventions for Pre-eclampsia (CLIP) (NCT01911494) [11]. This study was conducted in two districts, Matiari and Hyderabad, where 23 % of the population of Sindh province resides. Hyderabad is located on the east bank of the Indus River, and contains the second largest city in Sindh province, while Matiari is a rural district located 25 km north from Hyderabad. Over 90 % of the population is Muslim, and Sindhi is the main dialect. The provincial literacy rate is 40 % (25 % male and 15 % female), and nearly half of the population is engaged in the agricultural sector [12].

Data was collected through 33 focus group discussions and 26 one-to-one in-depth interviews. Purposive sampling was used during regular household visits and community activities to identify married women of reproductive age and male decision-makers,. Male decision-makers included husbands, teachers, religious leaders, and community leaders. Traditional Birth Attendants (TBA) were few in number and were identified through snowball sampling. Lady Health Workers (LHWs) and Lady Health Supervisors (LHS) were identified through the office of the National LHWs Programme. LHS were selected through contact lists and were recruited at their monthly meetings. Woman Medical Officers (WMO) were identified and approached during visits to health facilities in the study catchment areas.

Participants were considered eligible, if they expressed willingness to participate in the study and were available for at least 60 min. All study participation was voluntary, and the background and purpose of the study were explained prior to obtaining consent. Participant characteristics are enumerated in Table 1.

**Table 1:** Distribution of FGDs/IDIs

Stakeholder group	Data collection method	Number of FGD/IDI	Number of participants
Mothers	Focus group	19	173
Male decision-makers	Focus group	7	64
Lady health workers	Focus group	7	64
Lady health supervisor	Interview	10	10
Women medical officer	Interview	9	9
Traditional birth attendants	Interview	7	7

Focus group and interview guides were developed for set of a priori themes informed by the literature. The topics were related to care during pregnancy and its complications, perceived severity of hypertension and seizures during pregnancy, and community based practices in pregnancy. The themes included, knowledge and perceptions/beliefs/experiences about pregnancy associated common illnesses, perceptions and beliefs for prevention of pregnancy complications, delays in care seeking for the management of complications during pregnancy and cost of care (direct and in-direct). All focus groups and interviews were conducted in Sindhi or Urdu languages to best fit the needs of participants. To respect local preferences, focus groups were held separately for women and men at the local venues. Data saturation was confirmed though regular reviews of the focus groups and interviews conducted.

Each focus group was conducted by one facilitator, two note takers, one observer; and discussions were audio-recorded. Interviews were conducted by native Sindhi/Urdu speaking, gender specific staff, with a medical or social science background. Project staff were locally recruited and trained by a senior faculty member and a social scientist with first-hand knowledge and expertise in qualitative research. The field staff then developed local Sindhi languages transcriptions based on the recorded information. Reflexive notes were maintained for all FGDs and IDIs to capture field observations. Quality control was ensured through random observation of FGDs and IDIs by the field co-ordinator, and an audit-trail of 20 % of FGD transcripts. The audit-trail process included verifying content of transcripts with audio-recordings, and biweekly debriefing sessions with moderators/transcribers led by the senior project staff. Data were typed in Sindhi and reviewed to ensure that all the information had been recorded accurately. The data was analysed in Sindhi language using NVivo version 10 [QSR, Doncaster Vic, Australia] to develop the themes and subthemes from an ethnographic approach. After verbatim transcription in local language, the transcripts were translated into English, with back-translation to ensure data quality.

This study received ethical approval from Ethics Review Committee of Aga Khan University, Karachi Pakistan, National Bioethics Committee of Pakistan [1917-Obs-ERC-11] and Institutional Review Board of University of British Columbia, Vancouver Canada [H12-00132].

## Results

The findings from this study have been categorized in seven thematic areas. These include the women and men’s perspectives, as well as that of community-level care providers, LHW, TBA, and WMO.

### Theme one-Knowledge, perceptions, beliefs, experiences about common pregnancy complications

Most women claimed not to seek regular antenatal care (ANC); however, those that did followed-up as instructed by their health care provider. The perception was that ANC is only needed in the event of complications: *“If there is no problem then why should we go, but if there is some problem then one should go” [mother].* Women accessed ANC from public and private facilities.

### Theme two-Perceptions and beliefs for prevention of pregnancy complications

Majority of women stated *“Vertigo, headache, weakness and excessive bleeding are the reasons we visit doctor while we are pregnant”* as they are ‘danger signs’ or complications of pregnancy. Some women also included ‘*high blood pressure and seizures*’ as danger signs after probing. When asked directly all women knew the terminology of high blood pressure and, stated that high blood pressure can occur during pregnancy. Women also stated that this may lead to “*weakness, headaches and palpitations in the mother; child could be born weak” [mothers].* They further stated that they knew they had high blood pressure when they experienced headache, weakness, dizziness, pain in back and legs, and swelling of the feet. They did not state measurement of blood pressure as a way to identify the problem. When they experienced these symptoms they would self-medicate with drugs purchased over the counter.

Women believed that when these symptoms were severe, they should seek care in health facility.

Male decision-makers stated that weakness/anaemia and the lack of foetal movements were indications of a problem requiring a health care provider*: “Mostly there is anaemia” [male decision-makers].*

Community health care providers-LHS and TBAs - reported that it is a lack of knowledge and awareness about pregnancy complications that ultimately delay women from accessing services. LHS and TBAs claimed this deficient knowledge exists amongst pregnant women and their families. In addition, there were potentially harmful misconceptions held by women and families regarding the severity of pregnancy complications. Women stated “stress and poverty” as a cause for their raised blood pressure and did not state the connection between pregnancy and high blood pressure. This was further confirmed by the following statement *“People do not take blood pressure seriously; they think it will get better if they take rest and do not take salt”* [*LHS and TBAs*].

### Theme three - Delays in care seeking for the management of pregnancy complications

#### Barriers to care seeking in pregnancy

Several barriers were identified by women that hindered access to health care services. These barriers included lack of child care, poor access to transport, significant distance to facility, and the lack of a male chaperone. In some instances an elderly female took on the role of chaperone when men were not available.

*There are many problems; sometimes we have money sometimes we don’t. […] If husband is home he will take us otherwise we stay at home* (Mothers)

*The hospital is far away and our husbands are also not at home. It takes time to arrange transport. It is very difficult to reach the hospital on time* (Mothers)

#### Decision-making power as a determinant for care seeking in pregnancy

The majority of the respondents agreed that the principal decision-maker for health care is the husband: “*Obviously my husband decides”* [mother]. Some men preferred TBAs when their wives accessed services, because they believed that TBAs were readily available to deliver care at home at a decreased cost. These preferences influenced women’s care seeking due to the influential role of the husband. Nevertheless, in some cases the mother-in-law, with whom the woman is residing, will make decisions: *“We agree on what our husbands say, we also do what our mother-in-law says”* [mothers]. There were only a few women who stated that they were able to make the decision on their own*: “I myself decide, I visit doctors for medication when I am ill”* [mothers].

### Theme four - Cost and the perceived burden of care

Male decision-makers described that they took loans when money was needed for transport and health services. Such loans could be repaid in installments or by selling goods such as cattle:

*If any animal bull etc. is at home, we sell it in half price to return the money* [Male Decision-Makers].

### Theme five - The role of traditional and spiritual healers in pregnancy

The community claims not to be using alternative medicines or providers (homeopathic, ayurvedic or herbal). One mother described how these types of services are no longer chosen: “*People of our community don’t use any other alternative medicines because they don’t trust them, we take English medicines only”* [Mothers].

LHWs confirmed that allopathic medicine was preferred over homeopathic or herbal medicine in their community: *“Scientific medicines are used. No other”* [LHW]. However, there were home remedies which pregnant women take on recommendation by community elders and LHW themselves: *“For this salt intake can be decreased, oil intake can also be decreased, spicy foods intake can also be decreased. This is very beneficial”* [LHW].

Aside from these traditional practices, some men reported that women consulted ‘local religious leaders’ because of concerns of supernatural involvement. Faith-based treatments were described as an adjunct to formal health care services and not as a replacement.

*She should be given treatment, made to consult doctor. In home remedy, she is given massage on her head. It often occurs in rural areas. Spiritual healing is also done in order to see if she is under some supernatural influence. We take her there. The ‘faqir’(spiritual healer) treats through ‘jhaar, phoonk”(chanting and blowing on the person or afflicted part of the body) and also do ‘jhaar, phoonk’ on water and give her to drink. They also give ‘taveez’ (amulet) to wear. This is a practice in rural areas.*(Male Decision-Makers)

### Theme six - Community support during pregnancy

The family plays an important role in assisting women in pregnancy. Family mainly consists of the husband and the in-laws living in the same house or compound. Rarely family may include the natal members of the woman’s family. Respondents described the integral role family support plays in maternal health care service utilization. The family may assist in arranging transport or contributing funds*: “My husband and my mother arranged transport and took me to the hospital on the right time”* [Mothers].

The community may also play a role in assisting families to access health care services. In some cases where private vehicles are available in the neighborhood, this was used for transport to facility. In-kind support was also described by respondents, such as providing cooked meals. In spite of these examples, many women claimed the community was not involved in providing assistance: *“They don’t help. The community can’t do anything to save the woman”* [Mothers]*.*

### Theme seven - Preferred health care providers

In emergency cases, private health facilities were often preferred if the family can afford them. This preference was due to greater availability of physicians, timely care and availability of services. Private facilities were also preferred as they have a greater number of female providers, which is more culturally acceptable for care in pregnancy and postpartum. In the absence of adequate funds families were forced to patronize public facilities, where the quality of care is felt to be inferior - *“The people there are just not good, so we go to private. In government you also don’t know anything when checking the BP. They don’t do that well either”* [Mother]. In addition to the perceived low quality of services, some respondents stated that health care providers treat women poorly in public facilities: *“Private hospitals have more facilities and the attitude of doctors in government hospitals is also very rude”* [LHW]*.* Women complained that government facilities have long wait times: *“They have to stand in a long queue. The doctors give slips and make them stand for long hours. The doctor does not give them time and the condition of the woman gets worse”* [LHW]*.*

## Discussion

This study’s findings present the reported health seeking behaviour of rural women in Sindh Province, Pakistan as well as the barriers. This study reported that women and decision-makers are often not familiar with the importance of routine antenatal care. They believe that women should seek health services only when there is a complaint or complication. Women’s’ beliefs regarding antenatal care has been evaluated by Mumtaz et al [13], which found that most rural residents in the Punjab strongly believed that pregnancy is meant to lead to childbirth and hence should not be interfered with. This study also showed that villagers believed medical interventions during pregnancy can be harmful [13], and some symptoms are common and acceptable, and therefore did not seek care [14].

Headache is one such symptom that is commonly regarded as a sign of high blood pressure. Kruszewski et al. reported that in people with mild or moderate hypertension, headache was not significantly associated with raised blood pressure [15]. Further, Sperling et al. reported that headache as diagnostic criteria for preeclampsia is unreliable, nonspecific, and does not accurately predict adverse maternal and fetal outcomes [16]. The female participants in our study were also noted to report high blood pressure if they experienced a headache, without any objective measurements. Stress and family issues were commonly expressed by the participants as the cause of the headache and hence raised blood pressure.

Similar observations have been noted for other illnesses as well. Galloway et al. explored women’s perceptions of anemia from eight developing countries. It was noted that 90 % of women assumed that they had anemia based on the symptoms of weakness, headache, pale look and feelings of light headedness [17]. Most of these were the perception of the participants rather than objective measurements. This finding is consistent with our results as well.

The study results demonstrated a strong preference for private facilities for reasons that included availability of doctors, better equipment and facilities. Women’s perception of the quality of care available at the facility significantly influenced their choice [18]. Sheikh et al reported that in the northern Pakistan, the gender of health care providers, quality of service provided at the health facility and the associated financial cost were important factors in considering whom to consult [19]. Anwar et al also reported that in Pakistan, usage of public facilities was lower in rural areas due to restricted hours of operation, non-availability of drugs, distant locations and lack of female providers [20]. Women are frequently forbidden to visit the government facilities as there are mostly male doctors on duty [19]. It is unfortunate that this tendency also inhibits them from seeking care when resources are limited as private facilities are expensive. This perception and its associated health care behaviour are seen in many countries where public health systems are of low quality [21].

Results showed women generally are looked after and follow decisions of the husband and/or elder women in their community. Similar patterns of care seeking in pregnancy have been shown in rural Haiti [22] and in Bangladesh where the husband and mother-in-law were the main decision-makers for antenatal care [23]. A study done by Fatmi et al in rural Sindh concluded that husband’s educational status and occupation were significantly associated with utilization of antenatal care [24].

Resources are available within the immediate families and community to support women in accessing health care services. Social support from the husband, family or friends had the potential to influence care seeking behaviour. Social support in pregnancy is crucial for women in developed as well as developing countries [25, 26]. Mobility of some women is restricted, in some tribes this may be a self-imposed restriction i.e. ‘Brohi’ tribe in this study. Similar findings were reported by Mumtaz et al. where pregnant women avoided public places and travelling to access antenatal care due to the association of pregnancy with sexual activity [27]. The need for a companion is the norm in Pakistan, if the woman is admitted the companion is required to purchase any required materials or medications [13]. Women in Sindh use local resources to get help before approaching a health facility. Community resources such as TBAs as well as advice from the local pharmacists are utilized by pregnant women and their families. This phenomenon has also been observed in studies in Bangladesh where majority of women (68 %) purchased medicine from the store to use at home [28].

Finlayson et al.’s meta-synthesis of antenatal care services in low and middle income countries [29], reported that antenatal care was delayed because pregnancy was considered a healthy state which did not require intervention, access was further limited by financial constraints or distance. It was also reported that unprofessional attitude of the staff and unavailability of resources at the health facility further hindered health seeking [29]. Our study corroborates these findings. Further, it adds to the literature by exploring the community support available to the family in times of need as well as the use of alternative medicine during pregnancy.

The province of Sindh has a multi-ethnic population, which may not be fully represented in these results; however, the findings have shown similar themes across groups. Nevertheless, there may be subtle differences in the way these people perceive antenatal care which may have not surfaced in this study. Further, this study does not explore the deficiencies in the care process and infrastructure of the various health care facilities.

## Conclusion

This study revealed that health care has to be sought with the permission of and accompanied by a male family member or female elder in rural Sindh. Pregnancy was not considered a high-risk situation and therefore women presented infrequently for antenatal care. Women need to have clear concepts about symptoms that indicate emergencies during pregnancy and childbirth, as misconceptions regarding the severity of various conditions were reported. The community recognized that emergencies can arise during childbirth and in these cases women should access health services. Nevertheless, socio-cultural factors came into play causing delays. In addition, lack of high quality facilities at the public level prevented women from accessing appropriate and timely care.

There are policy implications for such findings. Firstly, it is important to educate the community regarding the benefits of regular antenatal care as well as the danger signs of pregnancy. LHWs should reinforce health and educational messages to women as well as the community during routine visits. Educational messages can be highlighted at primary health facilities along with radio or television messaging. It is also essential that the public sector ensure quality of services at all levels; this can be achieved by ensuring availability of qualified, competent, caring and preferably female staff at public and private facilities. Procedures and policies for management and referral of high risk obstetric cases need to be in place so that treatment delays are reduced. Improving the standard of care available to the pregnant women during emergencies is essential if maternal mortality is to be reduced.

### Peer review

Peer review reports for this article can be found in Additional file 1.

## Additional file

Additional file 1: Peer review reports. (PDF 616 kb)

## Abbreviations

ANC: antenatal care
CLIP: Community Level Interventions for Pre-eclampsia
LHS: lady health supervisors
LHW: lady health workers
MMR: maternal mortality ratio
PDHS: Pakistan Demographic and Health Survey
TBA: traditional birth attendants
TBA: traditional birth attendants
WHO: World Health Organization
WMO: woman medical officer
