# Supplementary material for: Health care seeking behaviours in pregnancy in rural Sindh, Pakistan: a qualitative study
Source: Reprod Health. 2016 Jun 8;13(Suppl 1):34. doi: 10.1186/s12978-016-0140-1 (PMC4943512; doi:10.1186/s12978-016-0140-1)
Supplement: Additional file 1: — Peer review reports. (PDF 616 kb) [file 12978_2016_140_MOESM1_ESM.pdf]

Referee's comments to the authors– this sheet WILL be seen by the author(s) and published with the article

Title	**Health care seeking behaviours in pregnancy in rural Sindh, Pakistan: a qualitative study**
Author(s)	Rahat Najam Qureshi, Sana Sheikh, Asif Raza Khowaja, Zahra Hoodbhoy, Zaidi Shujaat, Diane Sawchuck, Marianne Vidler, Zulfiqar A. Bhutta, Peter von Dadeslzen and the CLIP Working Group
Referee's name	Susan Dahinten

When assessing the work, please consider the following points, where applicable:

1. Is the question posed by the authors new and well defined?
2. Are the methods appropriate and well described, and are sufficient details provided to replicate the work?
3. Are the data sound and well controlled?
4. Does the manuscript adhere to the relevant standards for reporting and data deposition?
5. Are the discussion and conclusions well balanced and adequately supported by the data?
6. Do the title and abstract accurately convey what has been found?
7. Is the writing acceptable?

Please make your report as constructive and detailed as possible in your comments so that authors have the opportunity to overcome any serious deficiencies that you find and please also divide your comments into the following categories:

- Major Compulsory Revisions (which the author must respond to before a decision on publication can be reached)
- Minor Essential Revisions (such as missing labels on figures, or the wrong use of a term, which the author can be trusted to correct)
- Discretionary Revisions (which are recommendations for improvement but which the author can choose to ignore)

Where possible please supply references to substantiate your comments.

When referring to the manuscript please provide specific page and paragraph citations where appropriate.

GENERAL COMMENTS:

Although this study addresses an important global health issue – the need for a comprehensive understanding of factors affecting the utilization of antenatal health services – It is not clear how this study/manuscript adds to the literature. What is the critical gap in the literature that this study addressed? What were the key findings?

MAJOR COMPULSORY REVISIONS:

It is not clear what a priori themes informed the focus group and interview guides. (Also, some of the points outlined on page 7, lines 121-122, seem to be topics rather than themes.) Consequently it is not clear what, if any, additional themes emerged from the data. Please clarify.

Should clarify how this study/manuscript adds to the literature. For example, do the study findings provide any fresh insights compared with the meta-synthesis of Finlayson & Downe (2013)?

(continue on the next sheet)

Continued

Methods:

- Please describe the analytic process in more detail and procedures used to enhance rigor of analysis. Reflexivity would seem to be a particularly important aspect of this study, given that the research team started with a priori themes.
- Quotes are provided in English, but no information is provided regarding translation from the transcripts which were typed in Sindhi. I am particularly curious about some of the quotes included in regards to reasons for seeking care … e.g., page 8, line 158 "most frequently stated as high blood pressure, excessive bleeding and seizure". How would the women know that they had 'high blood pressure' prior to seeking care? Similarly, page 9, Lines 162-164 … "Male decision-makers stated that anemia and the lack of foetal movements were indications of a problem requiring a health care provider". How would the men know that the women were anemic? These quotes are also inconsistent with comments made by community health care providers that there is a lack of knowledge and awareness of pregnancy complications that delay women from accessing services (page 9 Lines 166-167). These issues may lead some readers to question the trustworthiness of the data.

Discussion/Conclusion:

- Implications of study findings and the authors' recommendations should be discussed in more detail. As currently written, the policy recommendations seem somewhat shallow given the resource constraints in LMICs and the complexities of the problem. Given the difficulties of making changes in the short term to the factors identified in this study that influence health care seeking, what specific approaches do the authors recommend at different time frames, i.e., in the short term, intermediate term, and long term? And how do you suggest these changes should be approached? Can you identify any practice implications/innovations? For example, how/who could influence the attitudes of decision-makers (husbands, mothers and mothers-in-law)?

MINOR ESSENTIAL REVISIONS:

The manuscript should be reviewed for syntax and punctuation (e.g., missing articles; non-parallel forms of nouns, verbs, and pronouns; the mis-use of commas vs semi-colons).

DISCRETIONARY REVISIONS:

Abstract:
The second sentence in the abstract (Lines 3 and 4) suggests that the sample consisted only of pregnant women who had made a decision to seek maternal care services, rather than also including pregnant women who may have decided against seeking maternal health care services.

Background:
(pages 3-5) should be re-organized for a better flow. Some additional detail/more specificity would also be helpful; for example, rather than stating that only 65% of women seek routine care during pregnancy, report % of pregnant women who access antenatal care per WHO standards.

Methods:
Page 7, Line 128-129. What was the background of the facilitators, note takers, and observers?

(continue on the next sheet)

Continued

Findings:

- Page 8, Line 145, notes that findings are categorized in five prominent themes, but seven themes are identified on pages 8 through 12.
- Some of the “five prominent themes” could be written more informatively (i.e., as thematic sentences) to more clearly respond to the research objective of understanding health seeking patterns of pregnant women in rural Sindh, Pakistan.
- Deeper analysis, or additional analysis to integrate the themes or explore their inter-relationships, would strengthen this study.

Discussion:

Could add some discussion/speculation on whether/which ‘barriers and facilitators’ found in this study are relevant more globally, or to health care seeking in general.

Referee's comments to the authors– this sheet WILL be seen by the author(s) and published with the article

Title	**Health care seeking behaviours in pregnancy in rural Sindh, Pakistan: a qualitative study**
Author(s)	Rahat Najam Qureshi, Sana Sheikh, Asif Raza Khowaja, Zahra Hoodbhoy, Zaidi Shujaat, Diane Sawchuck, Marianne Vidler, Zulfiqar A. Bhutta, Peter von Dadeslzen and the CLIP Working Group
Referee's name	Kate Teela

When assessing the work, please consider the following points, where applicable:

1. Is the question posed by the authors new and well defined?
2. Are the methods appropriate and well described, and are sufficient details provided to replicate the work?
3. Are the data sound and well controlled?
4. Does the manuscript adhere to the relevant standards for reporting and data deposition?
5. Are the discussion and conclusions well balanced and adequately supported by the data?
6. Do the title and abstract accurately convey what has been found?
7. Is the writing acceptable?

Please make your report as constructive and detailed as possible in your comments so that authors have the opportunity to overcome any serious deficiencies that you find and please also divide your comments into the following categories:

- Major Compulsory Revisions (which the author must respond to before a decision on publication can be reached)
- Minor Essential Revisions (such as missing labels on figures, or the wrong use of a term, which the author can be trusted to correct)
- Discretionary Revisions (which are recommendations for improvement but which the author can choose to ignore)

Where possible please supply references to substantiate your comments.

When referring to the manuscript please provide specific page and paragraph citations where appropriate.

General comments:

This paper does a good job at comparing findings to what else is in the literature, in Pakistan and elsewhere.

Major compulsory revisions:

Minor essential revisions:

General editing for grammar and missing words throughout is needed. A couple examples include:
* Line 46 – missing "they", Line 245 – missing "to", etc.
* Lines 63-65 – recommend consistency on use of numerals versus typing out numbers, % versus 'percent

Continued:
I think some quantification of results would be helpful. Phrases such as "most women…" are used often, but there is no quantification of what 'most' means. %s applied to these statements would greatly strengthen the arguments made. Also, without any sort of quantification, it is difficult to take statements such as "The community claims not to be using alternative medicines or providers" (line 189) seriously as it appears that this is just one quote from the study saying this.

Further detail on the methods would be helpful – How were all the staff members discussed trained (if quality control is reliant on them, this information is relevant)? Detail on the purpose of the observer during the focus groups would be helpful. Who conducted the debriefing sessions? Always the field coordinator? How were interviews conducted and how was data control maintained for the interviews (only the focus groups are discussed)? Some discussion on the implications behind the varying numbers of types of respondents would be helpful (e.g. very few TBAs compared with LHWs – is this reflective of the proportion in reality?, etc).

How was bias dealt with during analysis once the data was in Nvivo? Was there blind-coding done by another person?

Line 125 – how are you defining data saturation?

Lines 222-229 – How is it possible that "all respondents" said that the husband is the principal decision maker for health when there is a quotation given by a woman saying she decides for herself (line 229)?

Discretionary revisions:

Some of the references (e.g. reference #3) are quite old – it would strengthen the arguments made if there was a more recent reference.

Besides the need to improve quality at the public sector, what are the policy implications behind people preferring private sector? Are there lessons to be learned from the private sector, etc? Some explanation around why the paper recommendations are focused only on the public sector would be helpful.

Supplement Editor's comments:

Please have a look of the references. In some of them the volume is missing and please provide the date that you accessed the links.
